# Lung Cancer Surgery after Neoadjuvant Immunotherapy

**DOI:** 10.3390/cancers13164033

**Published:** 2021-08-10

**Authors:** Dirk Stefani, Till Plönes, Jan Viehof, Kaid Darwiche, Martin Stuschke, Martin Schuler, Clemens Aigner

**Affiliations:** 1 Department of Thoracic Surgery, University Medicine Essen–Ruhrlandklinik, Tueschener Weg 40, 45239 Essen, Germany; dirk.stefani@rlk.uk-essen.de (D.S.); till.ploenes@rlk.uk-essen.de (T.P.); jan.viehof@rlk.uk-essen.de (J.V.); 2 Department of Pneumology, University Medicine Essen–Ruhrlandklinik, Tueschener Weg 40, 45239 Essen, Germany; kaid.darwiche@rlk.uk-essen.de; 3 Department of Radiation Oncology, University Medicine Essen, Hufelandstrasse 55, 45147 Essen, Germany; martin.stuschke@uk-essen.de; 4 Department of Medical Oncology, University Medicine Essen, Hufelandstrasse 55, 45147 Essen, Germany; martin.schuler@uk-essen.de

**Keywords:** lung cancer surgery, neoadjuvant immunotherapy

## Abstract

**Simple Summary:**

Neoadjuvant immunotherapy is a novel approach for lung cancer patients in stages where curative intent treatment is possible. The rationale is a based on the idea that recognition by the immune system is activated by the entire tumor prior to surgical resection. Promising pathologic response rates have been reported and the impact on survival is currently investigated in ongoing studies.

**Abstract:**

In early-stage lung cancer, recurrences are observed even after curative resection. Neoadjuvant immunotherapy might be a promising approach to eliminate micrometastasis and to potentially reduce recurrence rates and improve survival. Early trials have shown encouraging rates of pathologic response to neoadjuvant therapy and have demonstrated that surgery can be safely performed after neoadjuvant immunotherapy with various agents and in combination with chemo-(radio)therapy. However, whether these response rates translate into improved disease-free survival rates and overall survival rates remains to be determined by ongoing phase III studies.

## 1. Introduction

**Figure 1:**
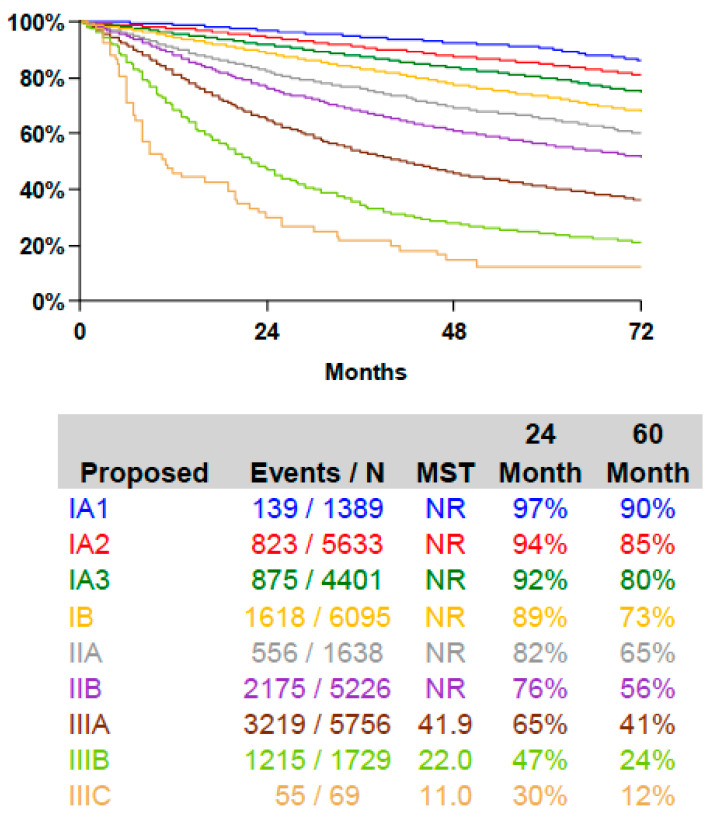
Survival after surgical resection of lung cancer according to TNM8 stage grouping [2].

Lung cancer is the leading cause of cancer death with a 5-year overall survival (OS) after diagnosis below 20% and is concurrently one of the most preventable cancer types [1]. Survival is clearly associated with the cancer stage [2] (Figure 1), and lung cancer screening in a high-risk population is able to reduce lung cancer related mortality [3,4]. Anatomical surgical resection with systemic mediastinal lymph node dissection is the mainstay of therapy in early-stage non-small cell lung cancer (NSCLC) [5] with adjuvant therapy in stage II and multimodal treatment regimens in stage III and oligometastatic stage IV. However, compared to other solid cancers, little progress has been achieved in improving survival rates in localized NSCLC patients throughout the previous decades. Thus, there is a need for additional treatment options. 

With promising results in the use of cancer immune modulators in advanced stage NSCLC patients, some focus has shifted to the use of immune checkpoint inhibitors (ICI) in early-stage NSCLC. The rationale for neoadjuvant ICI treatment is based on the hypothesis that the antigen load of the entire cancer might enhance immune recognition leading to a response of the primary tumor as well as potential micrometastases prior to surgical resection. This could be an advantage compared to adjuvant therapy. Studies exploring the use of neoajduvant immunotherapy or combinations of chemo-immunotherapy have shown the feasibility of this approach and promising results regarding pathologic response rates; however, the effect on disease-free survival (DFS) and OS remain to be elucidated in phase III studies. 

## 2. Brief Overview on the Current Status of Early-Stage Lung Cancer Surgery

Surgical resection is the most effective single modality and the primary treatment of choice for NSCLC patients in stage I and combined with adjuvant treatment in stage II. Minimally invasive techniques such as video-assisted thoracic surgery (VATS) or robotic-assisted thoracic surgery (RATS) are an established standard for lung cancer surgery and lead to a faster recovery and reduced perioperative morbidity [6] in the setting of enhanced recovery after surgery (ERAS) protocols [7]. Increased compliance with an ERAS pathway is shown to be associated with reduced morbidity, and early mobilization has been predictive of a shorter postoperative in-hospital length of stay [8] and lower occurrence of postoperative pulmonary complications [9]. Recent advances also include the use of VATS or robotic-assisted techniques in more complex cases [10,11,12,13]. The VIOLET study demonstrated comparable oncologic outcomes of the VATS approach to open techniques in a prospective randomized multi-center study with one year follow-up data currently being available [14]. In central and more extended tumors, multimodal treatment regimens are routinely established, and due to sleeve-resection techniques [15], the rate of pneumonectomies is low in experienced centers. In tumors < 2cm, data from the prospective randomized phase III Japan clinical oncology group (JCOG) 0802 study presented at the AATS 2021 [16,17,18] indicate a superior OS for segmentectomy compared to lobectomy. Since lung cancer related mortality was comparable in both groups, the results must be interpreted with caution until the full paper is available. If confirmed by other ongoing studies, this will lead to an extended use of parenchyma sparing sublobar resections with adequate lymphadenectomy in not only functionally compromised patients but as treatment of choice for T1a and T1b tumors as well. Multimodal treatment regimens are the standard of care in stage III and oligometastatic stage IV patients, and extended resections have become routine procedures in experienced departments. Additionally, the persistence of single lesions in stage IV patients on immunotherapy in a setting of mixed response has led to increased thoracic surgical consultation to evaluate resection of residual pulmonary lesions due to improved survival rates [19]. 

## 3. Neoadjuvant Immunotherapy in NSCLC

Programmed cell death protein 1 (PD-1) antibodies [20] as well as PD-L1 antibodies and cytotoxic T-lymphocyte-associated Protein 4 (CTLA-4) inhibitors [21] led to improved PFS and OS in patients with advanced or recurrent lung cancer [22,23] and are approved for routine use in NSCLC in various combinations. 

The mechanisms of the PD-1/PD-L1 and CTLA-4 pathways have been extensively studied for therapeutic checkpoint inhibition in NSCLC. Briefly, T cells express the surface protein PD-1 to regulate immune mechanisms, whereas tumor cells express PD-L1. Antigen presentation by the tumor cells is required to kill tumor cells by the inhibition of the PD-1/PD-L1 interaction. This antigen presentation activates host T cell who release cytokines after the blockade of PD-1/PD-L1 and kill the tumor cell [24]. The PD-L1 of the cancer cell and the PD-1 of the T cell can be blocked by antibodies. 

CTLA-4 is a glycoprotein expressed by both cluster of differentiation (CD)4+ and CD8+ T cells on their cell walls and are activated by tumor cells. Their activation leads to a direct inhibitory effect on the T cell expression. Furthermore, extrinsic CTLA-4 signals affect T cell adhesion to antigen-presenting cells (APCs), the T cell motility, and decrease residence time on the APC. Additionally, it prevents CD28, located in the T cell wall, from stimulating T cell proliferation or interleukin-2 production. In general, a hyperreaction of the T cell reaction is downregulated and prevented by CTLA-4. CD28 and CTLA-4 are opposing immune regulators [25]. CTLA-4, along with CD28, has been targeted by numerous therapeutic interventions.

The rationale for using neoadjuvant ICI treatment rather than adjuvant treatment is based on the idea that the antigen load of the entire tumor enhances the immune recognition and thus the efficacy of the therapy compared to an adjuvant application [26]. 

The assessment of the response for restaging after neoadjuvant immunotherapy has not yet been fully evaluated. Due to immune cell infiltration, there is a heterogeneity in published trials regarding whether tumor appearance on imaging correlates with pathological response.

## 4. Endpoints of Neoadjuvant Immunotherapy Trials

OS is the most important primary endpoint in the assessment of any neoadjuvant therapy [27]. However, in the neoadjuvant treatment of early-stage lung cancer patients, OS and DFS as endpoints require long follow-up periods. Thus, alternative endpoints that serve as surrogate parameters for survival have been investigated [27,28]. 

Radiologic and pathologic responses can be documented within a short timeframe after treatment. Objective response rate (ORR) on imaging including both partial and complete responses is an established endpoint for evaluating treatment effects after neoadjuvant chemo(-radio)therapy. However, there is heterogeneity in trials as to whether response imaging correlates to the pathological response due to the pseudoprogression of the primary tumor as well as the nodal immune flare. Thus, neither CT nor PET criteria for downstaging after neoadjuvant immunotherapy are currently established [26,29,30,31,32]. 

Major pathologic response (MPR) is defined as a residual viable tumor of less than or equal to 10% that has been established as a surrogate endpoint for survival after neoadjuvant chemotherapy leading to a shortened time required to evaluate neoadjuvant trials with novel chemotherapeutics [33,34,35,36,37]. Recently, MPR in any involved lymph nodes has also been shown to correlate to OS [38].

Pathologic complete response (PCR) of the primary tumor and the lymph nodes after classic neoadjuvant chemotherapy correlate to improved OS, but nodal response is dependent on the accuracy of nodal staging and is only applicable to patients with documented nodal disease [33].

The standardization of MPR evaluation standards has been controversial [39]; however, recently, a standard by the International Association for the Study of Lung Cancer (IASLC) for evaluating MPR after neoadjuvant therapy has been published with a recommendation for all neoadjuvant systemic therapies, including chemotherapy, chemoradiation, molecular-targeted therapy, and immunotherapy, alone or in combination [40]. The application of this standard should lead to clinical trials being comparable. 

The correlation of MPR and PCR with DFS and OS after neoadjuvant immunotherapy is however not yet known [27]. A considerable variability of pathologic response rates has been observed in previous phase II trials, which might be partially due to a lack of standardized criteria for pathologic evaluation prior to the IASLC recommendation (Table 1).

## 5. Re-Staging and Perioperative Management after Neoadjuvant Immunotherapy

### 5.1. Imaging Criteria for Restaging

Discrepancies between radiographic and pathologic responses occur after neoadjuvant immunotherapy, and there are conflicting data in published studies as to whether a correlation between imaging and pathologic responses is observed. Imaging can underestimate the degree of pathologic tumor regression [26] and persistent lung mass or adenopathy cannot definitively be assumed to represent residual active cancer, even if hypermetabolic on PET [32]. This is of particular importance after immunotherapy as a single neoadjuvant modality without the addition of chemotherapy. Despite a MPR rate of 45% in resected lung cancer after neoadjuvant nivolumab therapy, as described in the trial of Forde and colleagues, only two of twenty patients showed a partial radiologic response on presurgical CT scans, and the majority had stable disease. A total of two patients with an increased tumor size on a CT scan had minimal or no residual tumor. [26,31]. Thus, new algorithms for decision making to proceed with surgical resection after neoadjuvant immunotherapy will most likely be required and should be determined to be informed by the data of ongoing studies. 

Currently, no criteria as to when to proceed with surgery after complete radiological response after immunotherapy are established. A detailed assessment of CT and PET criteria to evaluate indicators of pathological downstaging should accompany neoadjuvant immunotherapy trials. An evaluation based on immune-related response criteria (irRC) rather than response evaluation criteria in solid tumor (RECIST) has been postulated [41]. An initial tumor expansion related to immune-mediated inflammatory response by immune cells infiltrating the tumor is also called a “tumor flare“ [42]. Radiographical disease progression in lymph nodes that are enlarged and possibly FDG avid, although histologically negative after ICI therapy, was named nodal immune flare (NIF) by the NEOSTAR trial group [43]. Nodal immune flare leads to a radiographic tumor upstaging and is likely due to T cell infiltration and not due to tumor cell infiltration. The immigration of T lymphocytes into the tumor tissue as TILs is part of the immune process after ICI therapy [41,44]. 

Algorithms to differentiate pseudoprogression from true progression or hyperprogression are required. Several biomarkers including PD-L1 status, tumor mutational burden, and baseline lymphocyte infiltration counts or immunoscore are under investigation; however, none have been established [45]. The importance of adequate sampling when assessing biomarkers is underlined by discordances observed between PD-L1 negativity in the initial biopsy compared to the histological result after surgery. The overall discordance rate was 48% with an underestimation of PD-L1 status on the initial biopsy in all cases. [46]. 

### 5.2. Biomarkers of Therapy Response

No biomarkers for therapy response have been established in clinical routine in the neoadjuvant setting. However, biomarkers are an important part in virtually all ongoing clinical trials, and a detailed discussion of all potential markers is beyond the scope of this review. Based on data from advanced stage NSCLC, PD-L1 and tumor mutational burden (TMB) [47,48,49] are among the most frequently investigated markers. 

TMB describes the number of mutations in cancer tissue. High TMB might be a predictive of response to ICI therapy in NSCLC and SCLC [50]. The LCMC3 trial used TMB as secondary endpoint. An interim analysis of this ongoing trial [51] showed encouraging pCR and MPR rates that were above a median TMB of 10.4 (range, 1.5-46.5) mutations per megabase. 

A high PD-L1 expression level could be identified as another independent biomarker [48] for prolonged OS in advanced NSCLC patients treated with PD-L1 antibodies [47]. In the phase II NADIM trial, a higher PD-L1 tumor proportion score was seen in patients who had a pCR than in patients with incomplete pathologic response (*p* = 0.042) [52]. However, in the phase II trial published by Shu and colleagues 2020 with neoadjuvant PD-L1 antibodies plus chemotherapy, no significant association between MPR or pCR and pretreatment PD-L1 status were observed [53].

In summary, the prognostic and predictive value of PD-L1, TMB, and other biomarkers for neoadjuvant immunotherapy has not yet been established. 

Circulating tumor DNA (ctDNA) after curative-intent first-line therapies might develop as a marker to detect residual disease and disease recurrence for lung cancer earlier than imaging. This was shown in more than 70% of the patients, with a median lead time of >5 months being highly prognostic in the detection of molecular residual disease. Detecting multiple mutations improved the sensitivity [54]. However, a lack of ctDNA detection after neodjuvant ICI was not a reliable marker for pCR [42]. CtDNA may best be assessed after surgery to identify patients with micrometastases and to select them for adjuvant therapy, with the aim of improving prognosis after surgery. 

### 5.3. Surgical Outcome after Neoadjuvant Immunotherapy

Complication rates, blood loss, procedure time, and perioperative course after neoadjuvant ICI therapy are so far comparable to patients receiving neoadjuvant chemotherapy or chemoradiation [19,55]. Initially, a higher conversion rate of minimally invasive to open procedures was reported; however, minimally invasive resections have been reported throughout published phase II trials and are feasible [56,57,58]. Nevertheless, frequently enlarged lymph nodes are observed and fibrotic adhesions, particularly at the hilum, but at the chest wall as well, might necessitate conversion from thoracoscopy to thoracotomy in some cases, and mediastinal and hilar dissection can be technically challenging due to inflammation or hypervascularity [59]. In the first published series of surgical results after neoadjuvant nivolumab therapy, 7 out of 13 attempted VATS cases (54%) had to be converted to thoracotomy due to technical challenges [57,58]. Dense fibrosis may develop as result of response to immunotherapy [32]. However, compared to neoadjuvant platinum-based chemotherapy, surgical resection rates and R0 resection rates are not inferior after neoadjuvant immunotherapy [52,55]. In perioperative management, a close collaboration between surgeons and oncologists is beneficial, particularly in the detection and management of the potential immunotherapy side effects [32]. 

### 5.4. Side effects of Immunotherapy

Immunotherapy has a different spectrum of side effects compared to other neoadjuvant therapies. These side effects can impact operability and affect the perioperative course. The increased use of immunotherapy in patients in a curative setting might lead to a more granular picture of possible therapy induced side effects compared to stage IV patients and might even highlight previously unknown long-term effects of immunotherapy due to prolonged survival times. Side effects mainly derive from the activation of the immune system and comprise a wide spectrum like pneumonitis, acute kidney injury, cardiotoxicity, hepatitis, dermatological reactions, neuropathy, nausea, muscular weakness, adrenal insufficiency, hepatitis, and gastroenteritis. [60].

Immune-related adverse events might arise immediately after therapy initiation; however, late onset months after the start of therapy has also been observed. Thus, onset can occur after the completion of the neoadjuvant therapy period, for example, in the early postoperative phase or during postoperative recovery [59]. Pneumonitis is of particular importance in patients undergoing lung surgery and might occur without compromising the respiratory situation, even months after therapy initiation [19]. In patients with a history of pneumonitis a course of steroids might be given perioperatively on an empirical basis [32]. In rare cases, pneumonitis might develop a fatal course despite a full escalation cascade of treatment, including corticosteroids and immunosuppressive medication including infliximab and cyclophosphamide [61]. 

Another severe adverse event is cardiotoxicity. A 0.06% myocarditis rate was observed after ICI treatment in patients with PD-1 antibodies with a higher incidence in patients receiving a dual ICI therapy together with a CTLA-4 inhibitor of 0.27%, shortly after application and partly after first dose administration [62]. Cardiotoxic events under ICI therapy are currently considered to be rare but may be serious and potentially fatal, especially with myocarditis and conduction disease with ventricular arrythmias and heart failure, but acute myocardial infarction and pericarditis may also occur [63]. A meta-analysis of 185 patients treated by neoadjuvant therapy with nivolumab, ipilimumab, atezolizumab, or sintilimab demonstrates a good tolerability with postoperative treatment related adverse events (TRAE) occurring in 12.5% of cases [55].

Awareness of the potential side effects and accurate diagnosis throughout the perioperative period is important to initiate appropriate treatment. 

## 6. Overview of Published Phase II Trials

Several randomized phase II studies comparing neoadjuvant immunotherapy or immuno-chemotherapy have been conducted. Some trial designs include adjuvant therapy as well [27]. These studies uniformly confirm the acceptable toxicity, morbidity, and mortality of neoadjuvant ICI therapies with or without chemotherapy [28]. Surgery after ICI therapy is considered to be safe regarding complication rates, blood loss, and operation time [19]. Table 1 summarizes the key facts of selected trials. 

Currently published trials were initiated when TNM7 was still effective. According to TNM, stage IB shifted to stage II in TNM8. This must be considered when interpreting trial outcomes. 

While a clinical paper on neoadjuvant chemoimmunotherapy in 2017 purely focused on immune profiling [44], the first trial on neoadjuvant immunotherapy alone was published by Forde in 2018 [31]. A total of two doses of nivolumab every two weeks in stage I-IIIA of NSCLC were used. In this single arm study of 22 enrolled patients, 20 received both doses of nivolumab, and while immune therapy related adverse events occurred in 5 patients (1 patient ≥ grade 3), no delay in surgery was observed. Upon imaging, partial response (PR) was seen in 10% of stable disease (SD) cases and in 85% of progressive disease (PD) cases in 5% of patients, however upon histopathology, MPR was seen in 45% of patients and PCR in 15% of patients. Surgery was more frequently performed by thoracotomy, otherwise surgical outcome was comparable to patients receiving other neoadjuvant treatments [57]. No outcome data beyond the perioperative period are available yet.

A phase II trial published by Shu and colleagues investigated i neoadjuvant atezolizumab and carboplatin/paclitaxel in patients with NSCLC stage IB-IIIA n an open label, multicenter, single arm setting [53]. No treatment related surgical delays were observed. Overall, 30 patients were included, and 29 were operated on. R0 resection was achieved in 26 patients. According to RECIST criteria, 63% of patients had a PR, 30% had a SD, and 7% had a PD. A post hoc analysis showed a significant association between RECIST criteria response and MPR, which was observed in 57%. pCR was seen in 33%. The most common treatment related adverse event was neutropenia, which was observed in 87% of all treated patients. No surgical complication attributable to neoadjuvant treatment were observed. 

The randomized NEOSTAR trial compared three cycles of neoadjuvant nivolumab with three cycles of nivolumab plus one cycle of ipilimumab in 44 operable stage I–IIIA NSCLC patients [43]. Out of these patients, 37 proceeded to surgery within the trial protocol, and 2 patients were operated outside the protocol. The R0 resection rate was 100%, and perioperative morbidity was within the expected range. ORR upon imaging was 19% in both groups, and a correlation with MPR rates was observed. MPR and pCR rates as well as the observation of enhanced tumor infiltrates and immunologic memory were higher in the nivolumab + ipilimumab combination group. Median DFS and OS were not reached in either arm after 22.2 months of follow-up. 

The IONESCO trial [64] investigating 3 courses of neoadjuvant durvalumab in stage IB-IIIA NSCLC stopped enrollment after inclusion of 50 patients. In 46 patients who proceeded to operation, an excess in 90-day postoperative mortality of 9% (4 patients) was observed. The deaths were due to postoperative complications that were possibly related to comorbidities and not to direct durvalumab toxicity [65].

The PRINCEPS [66] trial investigated a single dose atezolizumab as neoadjuvant therapy for stages IA (≥2 cm) to IIIA. Interim results presented as abstract showed the feasibility with all 30 patients proceeding to scheduled surgery; however, MPR was seen in only four patients (14%,) and no pCR was observed. No correlation between response in CT or PET and pathologic response was observed. 

The LCMC3 trial looked at MPR after two cycles of atezolizumab in stage IB- selected IIIB patients [67]. MPR was 21%, pCR 7%, and the therapy showed no safety signals. In 181 patients, 88% proceeded to surgery within the scheduled timeframe. The R0 resection rate was 92%, and low perioperative morbidity and mortality was observed.

Interim safety results of the NEOMUN trial [68,69] reported on the safety and feasibility of two cycles of neoadjuvant pembrolizumab in stage II/IIIA NSCLC. So far, 15 patients have been included, 13 (86.6%) have received the intended two cycles, grade 2-3 treatment related adverse events have been observed in 33%, and all patients have proceeded to surgery with a postoperative morbidity of 7%. 

The TOP 1501 [70] trial looks at two cycles of neoadjuvant pembrolizumab combined with four cycles adjuvant treatment in stage IB to IIIA patients and is ongoing. 

Only a few studies have focused exclusively on stage III patients. The NADIM trial investigated neoadjuvant chemoimmunotherapy with nivolumab and paclitaxel/carbolatin in a single arm design in stage IIIA (N2) patients followed by adjuvant intravenous nivolumab monotherapy for one year every two to four weeks [52]. Surgery was performed in 41 out of 46 included patients, and 37 received at least one cycle of adjuvant nivolumab. Adverse events ≥ grade 3 were observed in 14 patients (40%) during neoadjuvant treatment and in 7 (19%) during adjuvant treatment. According to RECIST criteria 2, patients (4%) had a complete response, 33 patients (72%) had a PR, and 11 (24%) had a SD. No PD was observed. Upon histopathology, 83% of the evaluated patients showed a MPR, and 63% showed a PCR. PFS at 24 months follow-up was 77.1%, and OS was 89.9%. 

The SAKK 16/14 trial [71] included 68 patients with stage IIIA(N2) NSCLC. Neoadjuvant treatment consisted of three cycle cisplatin/docetaxel followed by two cycles of durvalumab, which was continued after surgery biweekly for one year. Resection was performed in 55 patients. Radiographic response was 43% after chemotherapy and 58% after additional immunotherapy. MPR was 62% and pCR 18%. A total of 88% of patients experienced an adverse event that was of a grade ≥ 3, including two fatal adverse events that were judged not to be treatment related. The 1 year event-free survival (EFS) rate was 73% (two-sided 90% CI, 63 to 82). Median EFS and overall survival were not reached after 28.6 months. 

Interim data on 14 patients of the ongoing ACTS 30 trial investigating neoadjuvant chemoradiotherapy and durvalumab in potentially resectable stage III NSCLC were presented at ASCO 2021 [72]. Neoadjuvant therapy consisted of carboplatin/paclitaxel, 45Gy radiotherapy, and two cycles of durvalumab. Surgery was performed in eleven patients, and two are currently awaiting surgery. No postoperative in-hospital mortality was observed. MPR was 72.2%, and pCR was 36.4%. When patients with the EGFR mutation were excluded, the MPR raised to 88.0%. 

Several additional trials are in progress with no data yet published. A phase II trial comparing ICI alone and ICI with platinum-based chemotherapy in a neoadjuvant setting is the ongoing NAJSCR trial for resectable lung cancer treated with the new PD-1 antibody toripalimab [73]. The feasibility of preoperative immunotherapy with nivolumab combination therapies is determined in the ongoing investigator initiated randomized phase II NEOpredict trial for resectable Stage II-IIIA lung cancer patients [74]. The randomized ESPADURVA trial for stage III patients is based on the ESPATÜ protocol [75,76] and is concurrently exploring the PD-L1 antibody durvalumab as neoadjuvant therapy as well as adjuvant maintenance [77].

## 7. Ongoing Phase III Studies

KEYNOTE-671 [78], IMpower-030 [79], AEGEAN [80], Checkmate 77T [81], and CheckMate 816 [82] are randomized phase III trials assessing the efficacy of immunotherapy in combination with platinum-based chemotherapy in the neoadjuvant settings. 

In a first presentation of the trial results during the Annual meeting of the American Association of Cancer Research (AACR) held virtually on April 10th 2021, it was determined that nivolumab plus chemotherapy shows statistically significant improvement compared to neoadjuvant platinum-based chemotherapy alone in the pathologic complete response as a neoadjuvant treatment for resectable non-small cell lung cancer in the ongoing phase III CheckMate 816 trial by 24% versus 2.2% (*p* < 0.0001) and a MPR rate of 36.9% versus 8.9%, respectively. The benefit expands across the key subgroups: disease stage (IB/II of 26.2% versus 4.8%; ≥IIIA of 23% versus 0.9%), PD-L1 status (<1% of 16.7% versus 2.6%; ≥1% of 32.6% versus 2.2%), and TMB (low of 22.4% versus 1.9%; high of 30.8% versus 2.7%) [82,83]. Neoadjuvant nivolumab + platin-based doublet chemotherapy and adjuvant nivolumab versus neoadjuvant chemotherapy alone is investigated EFS as primary endpoint in the Checkmate 77T trial. Nivolumab versus observation after surgery and chemotherapy is under investigation in the ANVIL trial [84]. 

Pembrolizumab in the neoadjuvant setting is under investigation in the KEYNOTE-671 trial [78]. The endpoints are EFS and OS. Pembrolizumab with or without chemotherapy versus chemotherapy alone is currently tested also being investigated in the adjuvant setting in the KEYNOTE-091 trial (PEARLS) in stages IB–IIIA [85].

Atezolizumab plus chemotherapy versus chemotherapy alone is currently being explored in the neoadjuvant setting, and atezolizumab versus placebo is being investigated in the adjuvant setting for stages IB to IIIB (cT3 N2) in the IMpower 030 trial [79], the endpoints are MPR and EFS. Atezolizumab versus observation only after surgery and chemotherapy is under investigation in the IMpower010 trial [86]. IMpower110 was published in 2020 and showed significantly longer OS than platinum-based chemotherapy for both non-squamous and squamous-cell histologies for atezolizumab as a first-line treatment for patients with metastatic NSCLC with a PD-L1 expression status ≥1% [47].

Both durvalumab and chemotherapy versus chemotherapy neoadjuvant and durvalumab or placebo adjuvant is currently being tested both in the AEGEAN trial [80] with the endpoints of MPR and EFS, with durvalumab versus placebo as maintenance therapy in the CCTG BR31/IFCT-1401 trial [87] for resectable stages except for stage IA. 

Phase III trials of adjuvant immunotherapy or chemo-immunotherapy compared to neoadjuvant immunotherapy or chemo-immunotherapy do not yet exist; however, trials in this area would be warranted [27].

## 8. Future Perspectives

Depending on the long-term results of the ongoing phase III trials, immune checkpoint inhibitors might become part of standard therapy for localized NSCLC [32]. However, several open questions remain. Most trials are currently recruiting, with insufficient differentiation between stage II and stage III. The best sequence of immunotherapy and surgery, the best time interval between immunotherapy and surgery [19], whether to combine with chemotherapy and/or radiotherapy [28], whether a differentiated stage dependent approach is required, and the best way of integrating biomarkers into the selection algorithms are questions that need to be addressed by ongoing and future clinical trials. The issue of continuing with adjuvant therapy after neoadjuvant immunotherapy remains open as well as the correlation of pathological response parameters of MPR and pCR with clinical outcome [55]. Until now data have not been not available in sufficient granularity. 

Blood or exhalate based biomarkers with sufficient prognostic or predictive power have not yet been sufficiently evaluated in the neoadjuvant setting. TMB testing may complement PD-L1 testing in identifying patients who are likely to have good outcomes with immunotherapy [50]. 

Novel targets for immunotherapy are currently being explored. As an example, lymphocyte activation gene 3 (LAG-3), an immune regulatory molecule for the T cells is being used in the NEOPREDICT-Lung trial. LAG-3 is expressed only on TILs and correlates with the expression of PD-1 and PD-L1 and might be related to tumor recurrence and prognosis [88]. LAG-3 has also been investigated as a surrogate marker for the durable clinical benefit of immune checkpoint inhibition [89]. 

Interdisciplinary cooperation and active participation or thoracic surgeons in the design and implementation of neoadjuvant ICI trials is crucial for the development of optimal strategies for patients with early stage NSCLC [28]. The outcomes of these trials will heavily depend on surgical expertise, and meticulous patient selection is important. Translational research should accompany all trials and will provide a better insight in the mechanisms of action of immunotherapy and in markers and parameters that impact response to therapy and clinical outcome [56]. 

## 9. Conclusions

Neoadjuvant ICI therapy in lung cancer treatment might be a promising approach to reduce recurrence rates and to improve outcome for early-stage lung cancer patients. To draw any definitive conclusions results of ongoing phase III trials have to be awaited. Available data show that surgery after ICI treatment can be conducted safely. There is a considerable rate of pathologic responses after neoadjuvant immunotherapy and an even higher response after neoadjuvant immuno-chemotherapy. How these results translate into DFS and OS remains to be seen. Interdisciplinary cooperation with the active involvement of thoracic surgeons is crucial for the development of optimal strategies for patients with early-stage NSCLC and provides an excellent opportunity for translational research. 

## Figures and Tables

**Table 1:** Histopathologic response rates in neoadjuvant immunotherapy trials.

Trial	Patients (*n*)	Neoadjuvant Treatment	Stage (TNM7)	CR	PR	SD	PD	MPR	PCR	DFS	OS
Forde	22 (20 operated)	2× Nivolumab	I–IIIA	0%	10%	85%	5%	45%	15%	NR	NR
LCMC3	101	2× Atezolizumab	IB–IIIB	43%		38%	19%	21%	7%	18 months 79% stage I/II 77% stage III	18 months 91% stage I/II 87% stage III
NADIM	46 (41 operated)	3× Nivolumab + Paclitaxel/ Carboplatin	IIIA (N2)	4%	72%	24%	0%	83%	63%	2y 77.1%	2y 89.9%
NEOSTAR (Arm A)	23 (21 operated)	3× Nivolumab	I–IIIA	19%		81%		24%	10%	Median not reached (22.2 months follow-up)	Median not reached (22.2 months follow-up)
NEOSTAR (Arm B)	21 (16 operated)	3× Nivolumab + 1× Ipilimumab	I–IIIA	19%		81%		50%	38%	Median not reached (22.2 months follow-up)	Median not reached (22.2 months follow-up)
Shu	30 (29 operated)	4× Atezolizumab + Paclitaxel/ Carboplatin	IB–IIIA	0%	63%	30%	7%	57%	33%	Median 17.9 months	Median not reached (12.9 months follow-up)
SAKK 16/14	68 (55 operated)	3× Cisplatin/Docetaxel 2× Durvalumab +adjuvant Durvalumab	IIIA (N2)	58% (43% after chemo alone)		42%		62%	18%	1 year EFS 73% Median EFS and OS not reached after 28.6 months	
Checkmate 816 (Arm A)	179	3× Nivolumab + platin based chemotherapy	IB–IIIA	NR	NR	NR	NR	36,9%	24%	NR	NR

CR: Complete Response, PR: Partial Response, SD: Stable Disease, PD: Progressive Disease, MPR: Major Pathological Response, DFS: Disease-Free Survival, OS: Overall Survival, NR: not reported, EFS: Event-Free Survival.

## Abbreviations

CD	Cluster of Differentiation
CR	Complete Response
ctDNA	circulating tumor DNA
CTLA-4	Cytotoxic T-Lymphocyte-Associated protein 4
DFS	Disease-Free Survival
EFS	Event-Free Survival
ERAS	Enhanced Recovery After Surgery
ICI	Immune Checkpoint Inhibitor
LAG-3	Lymphocyte-Activation Gene 3
MPR	Major Pathological Response
NSCLC	Non-Small Cell Lung Cancer
ORR	Objective Response Rate
OS	Overall Survival
pCR	Pathological Complete Response
PD	Progressive Disease
PD-1	Programmed cell Death protein 1
PD-L1	Programmed Death Ligand 1
PFS	Progression-Free Survival
PR	Partial Response
RATS	Robotic-Assisted Thoracic Surgery
SCLC	Small Cell Lung Cancer
SD	Stable Disease
TILs	Tumor Infiltrating Lymphocytes
TMB	Tumor Mutational Burden
VATS	Video-Assisted Thoracic Surgery
